# Primary Small Cell Carcinoma of the Thyroid With Absent Calcitonin Expression: A Diagnostic Challenge

**DOI:** 10.1111/pin.70204

**Published:** 2026-11-09

**Authors:** João Carlos Almeida, Patrícia Andrea‐Ferreira, Catarina Costa, Pedro Amoroso Canão, Elisabete Rodrigues, Manuel Sobrinho‐Simões, José Manuel Cameselle‐Teijeiro

**Affiliations:** ^1^ Department of Pathology Unidade Local de Saúde (ULS) São João (CHUSJ) Porto Portugal; ^2^ Faculty of Medicine of the University of Porto (FMUP) Porto Portugal; ^3^ General Surgery Department Unidade Local de Saúde (ULS) São João (CHUSJ) Porto Portugal; ^4^ Endocrinology Department Unidade Local de Saúde (ULS) São João (CHUSJ), ENDO‐ERN Porto Portugal; ^5^ Ipatimup (Institute of Molecular Pathology and Immunology of the University of Porto) Porto Portugal; ^6^ Department of Pathology, Clinical University Hospital of Santiago de Compostela, Galician Healthcare Service (SERGAS) Health Research Institute of Santiago de Compostela (IDIS), Medical Faculty, University of Santiago de Compostela Santiago de Compostela Spain

**Keywords:** carcinoma, immunohistochemistry, neuroendocrine, neuroendocrine tumors, thyroid neoplasms

## Abstract

Primary small cell carcinoma of the thyroid is an exceedingly rare and aggressive neuroendocrine malignancy. Diagnosis may be particularly challenging in tumors lacking calcitonin expression, in which distinction from medullary thyroid carcinoma and metastatic neuroendocrine carcinomas is required. A 50‐year‐old woman presented with progressive hoarseness and a rapidly enlarging cervical mass. Imaging revealed a highly infiltrative thyroid tumor with multiple cervical lymphadenopathies and no evidence of a primary tumor elsewhere. Serum calcitonin levels were within normal limits. Fine‐needle aspiration biopsy of the thyroid lesion and a cervical lymph node demonstrated cytologic features consistent with small cell carcinoma. Following a good response to neoadjuvant chemotherapy, total thyroidectomy with central, bilateral mediastinal lymphadenectomy was performed. Histologic examination demonstrated a highly infiltrative neoplasm composed of small cells arranged in solid and nested patterns with marked mitotic activity, tumor necrosis, and vascular and perineural invasion. Tumor cells expressed neuroendocrine markers with focal positivity for TTF‐1/NKX2‐1 and CEA, while calcitonin, thyroglobulin, PAX8, and CGRP were negative. Primary thyroid small cell carcinoma is exceptionally rare and its histogenesis remains uncertain. Diagnosis requires careful clinicopathologic correlation and exclusion of metastatic disease and calcitonin‐negative variants of medullary thyroid carcinoma.

## Graphical abstract

Primary small cell carcinoma of the thyroid is an exceptionally rare neuroendocrine malignancy with significant diagnostic challenges. We report a calcitonin‐negative thyroid neuroendocrine tumor with small cell morphology, absence of amyloid deposition, diffuse expression of neuroendocrine markers, no immunoreactivity for CGRP, and no RET/RAS mutations. Based on the morphologic, immunohistochemical, molecular, and clinical findings, a diagnosis of primary small cell carcinoma of the thyroid was established.

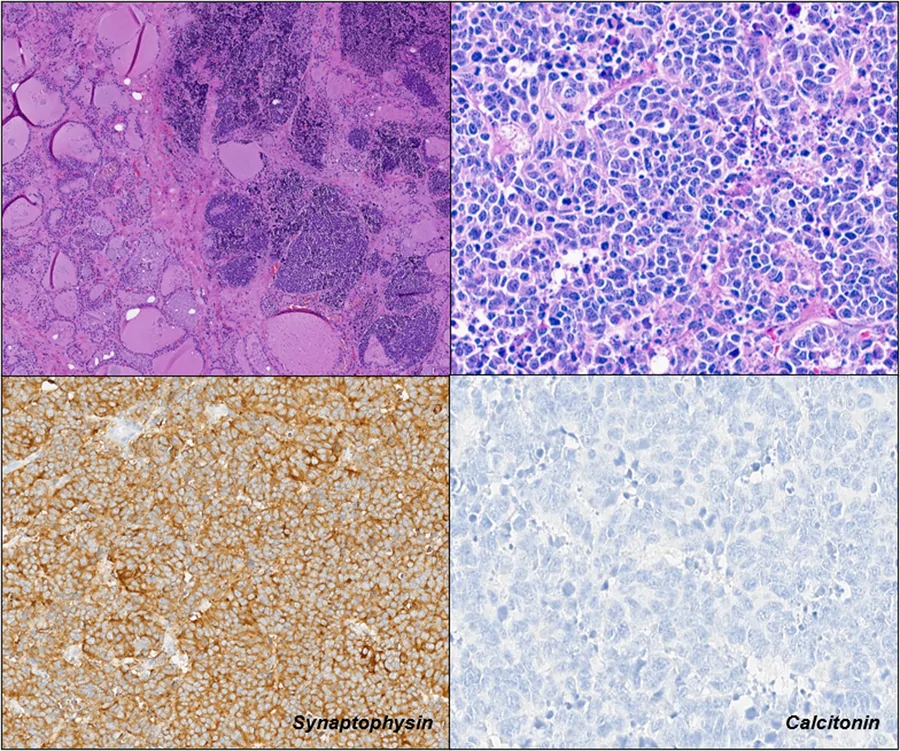

**Abbreviations**
CEA: carcinoembryonic antigen
CGRP: calcitonin gene‐related peptide
CK8/18: Cytokeratin 8/18
CK20: Cytokeratin 20
CT: calcitonin
EBER: Epstein–Barr virus‐encoded RNA
FNAB: fine‐needle aspiration biopsy
MTC: medullary thyroid carcinoma
SCC: small cell carcinoma
TTF‐1/NKX2‐1: thyroid transcription factor‐1

## 1 Introduction

Medullary thyroid carcinoma (MTC) is a malignant neuroendocrine tumor arising from the parafollicular C cells of the thyroid. Similar to normal parafollicular C cells, MTC cells synthesize and secrete calcitonin (CT), which can be measured in serum and is widely used for diagnosis, postoperative surveillance, and prognostic assessment [1]. In addition to elevated CT levels, carcinoembryonic antigen (CEA) may also be increased and can aid in the preoperative diagnosis of MTC [1].

Histologically, MTC most commonly exhibits solid, nested, or trabecular growth patterns composed of polygonal or round cells, frequently associated with variable stromal amyloid deposition. Less common morphologic variants include follicular, spindle cell, melanotic, and small cell patterns. In most cases, the histologic diagnosis of MTC is straightforward, as tumor cells demonstrate immunoreactivity for CT [2]. Molecular testing may further support the diagnosis, particularly through identification of *RET* or *RAS* gene mutations, which are detected in approximately 50%–80% of cases [2].

A small subset of MTCs lacks both serum CT elevation and immunohistochemical expression of CT [2]. In these situations, the main differential diagnoses include CT‐negative MTC, metastatic neuroendocrine tumors involving the thyroid, and primary small cell carcinoma (SCC) [3].

Primary SCC of the thyroid is an exceedingly rare and highly aggressive entity, with only a limited number of cases reported in the literature. Herein, we report a case of primary thyroid SCC and review previously published cases, highlighting the diagnostic challenges associated with this rare tumor.

## 2 Clinical Summary

A 50‐year‐old woman presented with hoarseness associated with a progressively enlarging cervical mass over a 1‐month period. The patient was HIV‐positive, had a history of multiple prior viral infections, and was an active smoker. Her past medical history was significant for chronic obstructive pulmonary disease. There was no known personal or family history of malignancy.

Physical examination revealed a large palpable mass in the thyroid isthmus region. Thyroid ultrasound demonstrated an asymmetrical gland with multiple hypoechoic solid nodules involving the isthmus and left lobe (ACR TI‐RADS 4), the largest measuring about 70 mm in greatest dimension, as well as multiple bilateral cervical lymphadenopathies. Serum CT levels and thyroid function tests were within normal reference ranges.

Fine‐needle aspiration biopsy (FNAB) of the cervical mass and a level III cervical lymph node showed findings consistent with involvement by SCC.

The patient underwent extensive staging evaluation. Flexible bronchoscopy demonstrated partial obstruction of the tracheal lumen due to anterior wall invasion by an exophytic lesion. Preoperative whole‐body computed tomography (Figure 1A) revealed a highly infiltrative cervical mass centered in the thyroid gland, invading the brachiocephalic trunk and left brachiocephalic vein, measuring up to 10 cm in greatest dimension, and associated with multiple lymphadenopathies. No primary pulmonary tumor mass or other primary neoplasm was identified at initial staging. A Gallium‐68‐DOTATOC positron emission tomography/computed tomography scan confirmed the aforementioned findings and supported the neuroendocrine nature of the tumor. Given the extent and infiltrative nature of the disease, the tumor was considered unresectable, and the patient received neoadjuvant chemotherapy with cisplatin and etoposide for 3 months. After completion of four cycles, imaging demonstrated a substantial reduction in tumor volume and decreased local mass effect, allowing surgical management. A total thyroidectomy with central and bilateral lymph node dissection, together with mediastinal lymphadenectomy, was performed.

**Figure 1:**
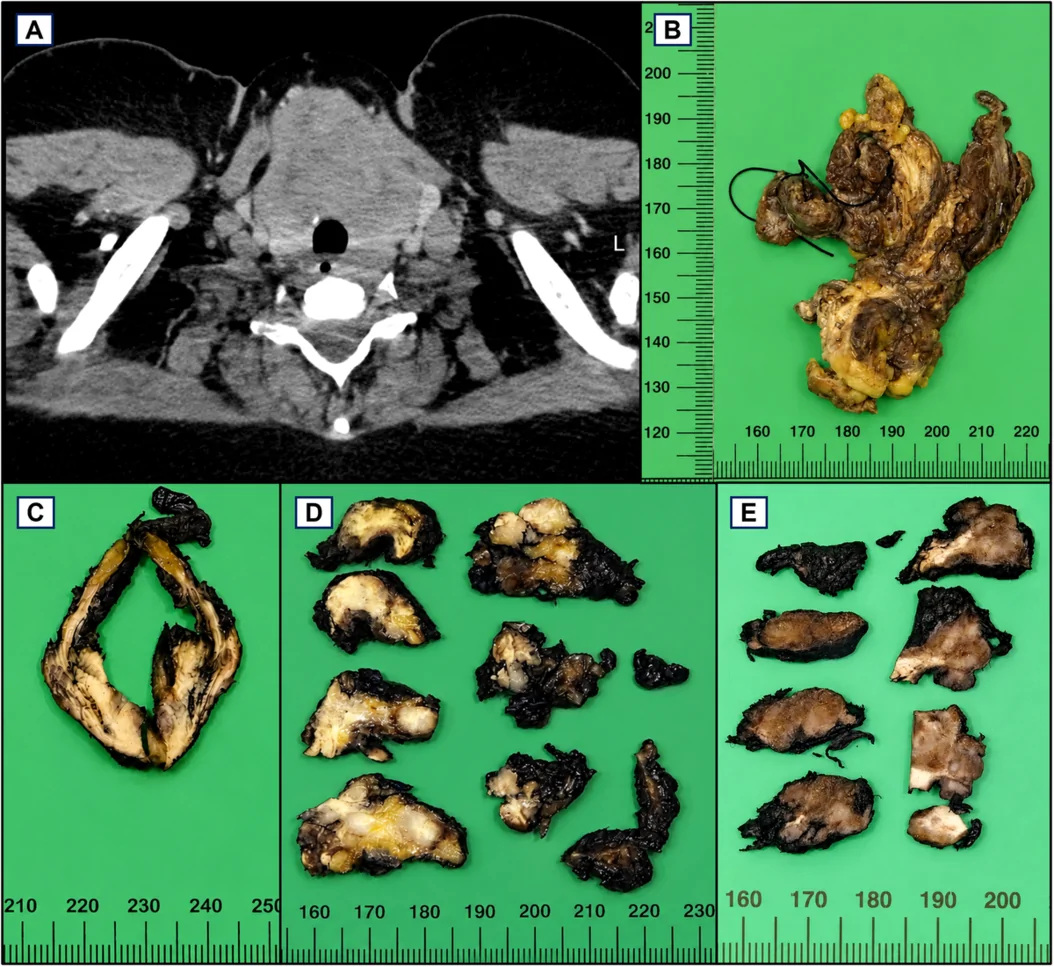
Radiologic and gross findings. Preoperative axial computed tomography demonstrates a large infiltrative cervical mass centered in the thyroid gland, with mass effect on the tracheal lumen (A). Gross examination of the thyroidectomy specimen reveals a markedly distorted thyroid gland (B). On sectioning, multiple white‐yellow nodular lesions are identified, largely replacing the thyroid parenchyma and extending into adjacent soft tissue, as illustrated in vertical sections through the isthmus (C) and horizontal sections through the central neck dissection tissue (D), and the left thyroid lobe (E).

Histopathologic examination confirmed the diagnosis of SCC. Radiotherapy was proposed but could not be completed due to patient intolerance. Three months after surgery, follow‐up computed tomography imaging demonstrated local tumor recurrence at the surgical site, newly developed lymphadenopathies, a small pulmonary nodule in the left lung, and multiple millimetric hypodense hepatic nodules. The pulmonary and hepatic lesions were clinically consistent with metastatic disease, although histologic confirmation was not obtained.

Chemotherapy with cisplatin and etoposide was resumed in the setting of progressive clinical deterioration; however, the patient died 10 months after surgery with locally recurrent and disseminated disease.

## 3 Pathological Findings

The initial diagnostic approach consisted of FNAB of the thyroid mass and of a cervical lymph node. Cytologic smears from both sites were hypercellular and composed of neoplastic cells with a high nuclear‐to‐cytoplasmic ratio, finely granular chromatin, nuclear molding, frequent mitoses, and numerous apoptotic bodies (Figure 2).

**Figure 2:**
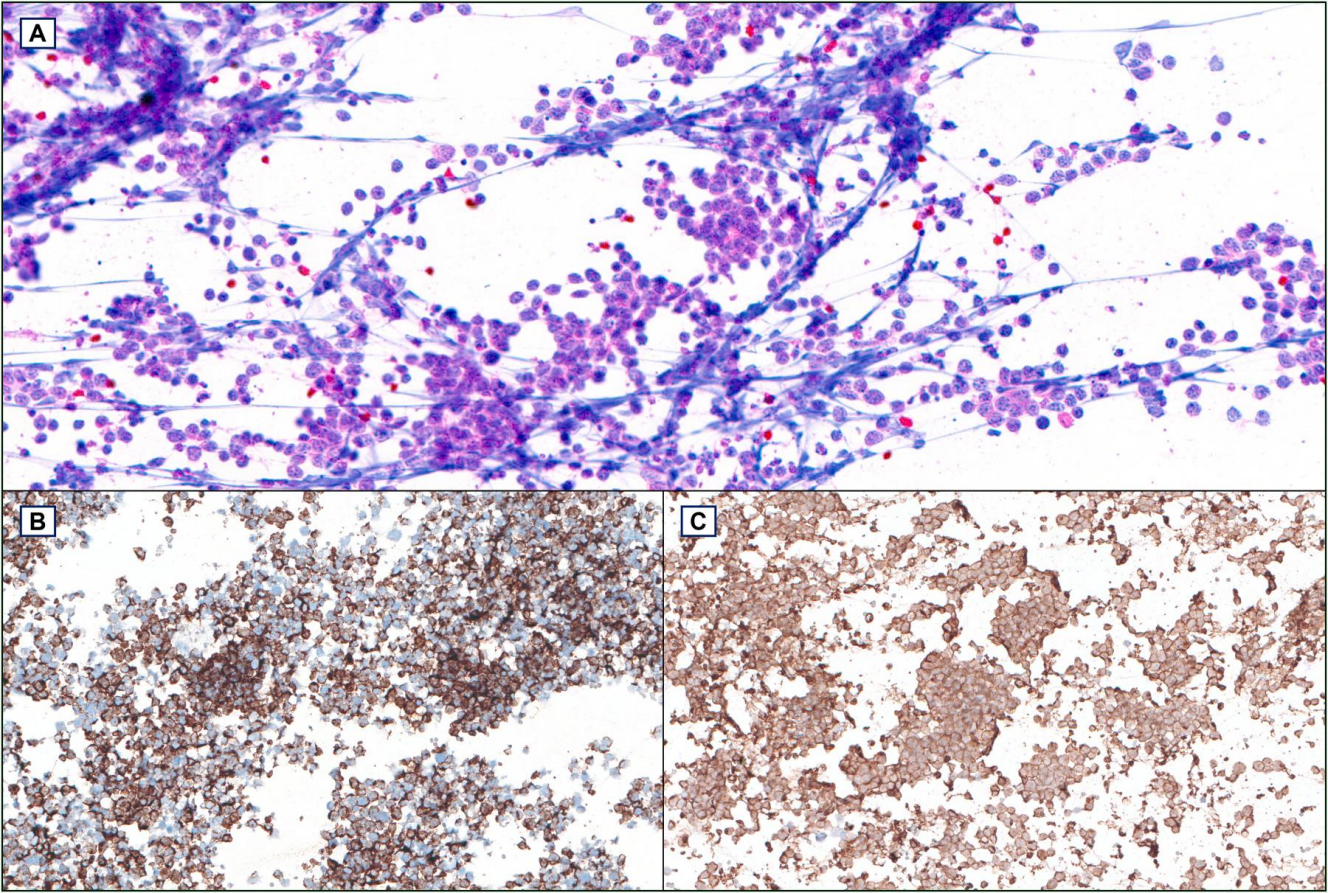
Cytologic findings from fine‐needle aspiration biopsy of the thyroid mass. Hypercellular smear composed of neoplastic cells with a high nuclear‐to‐cytoplasmic ratio, finely granular chromatin, nuclear molding, and frequent mitoses (A, hematoxylin and eosin stain, ×400). Immunocytochemical study demonstrated positivity for pancytokeratin AE1/AE3 (B, ×200) and the neuroendocrine marker synaptophysin (C, ×200) and CD56, supporting the diagnosis of small cell carcinoma.

An extensive immunohistochemical panel was performed, demonstrating positivity for pancytokeratin AE1/AE3, CD56, and synaptophysin, and negativity for PAX8, thyroglobulin, CT, p63, p40, p16, NUT, CK20, EBER (in situ hybridization), CD3, and CD20. These findings supported the diagnosis of SCC involving the thyroid and cervical lymph nodes.

The surgical specimen consisted of a markedly distorted thyroid gland weighing 16 g, with the right and left lobes measuring 3.2 and 4.0 cm in greatest dimension, respectively. On sectioning, multiple white to yellowish nodular lesions were identified, largely replacing the thyroid parenchyma, extending into adjacent soft tissue, and involving a parathyroid gland (Figure 1B–E). Only minimal residual thyroid parenchyma was preserved.

Microscopic examination revealed extensive infiltration of the thyroid gland and adjacent soft tissues by a neoplasm arranged in solid and nested growth patterns, with multifocal folliculotropism. The tumor was composed of neoplastic cells with cytologic features similar to those observed in the FNAB samples. Frequent areas of tumor necrosis, vascular and perineural invasion, and histologic evidence of treatment effect involving a substantial proportion of the tumor bed, characterized by fibrosis and a lymphohistiocytic inflammatory infiltrate with frequent foamy macrophages, were identified. (Figure 3). These findings were considered in keeping with the radiologic response to neoadjuvant chemotherapy. Although treatment‐related changes limited global mitotic assessment, up to 11 mitoses per high‐power field were identified in viable tumor areas. No amyloid deposits were identified (Congo red stain negative).

**Figure 3:**
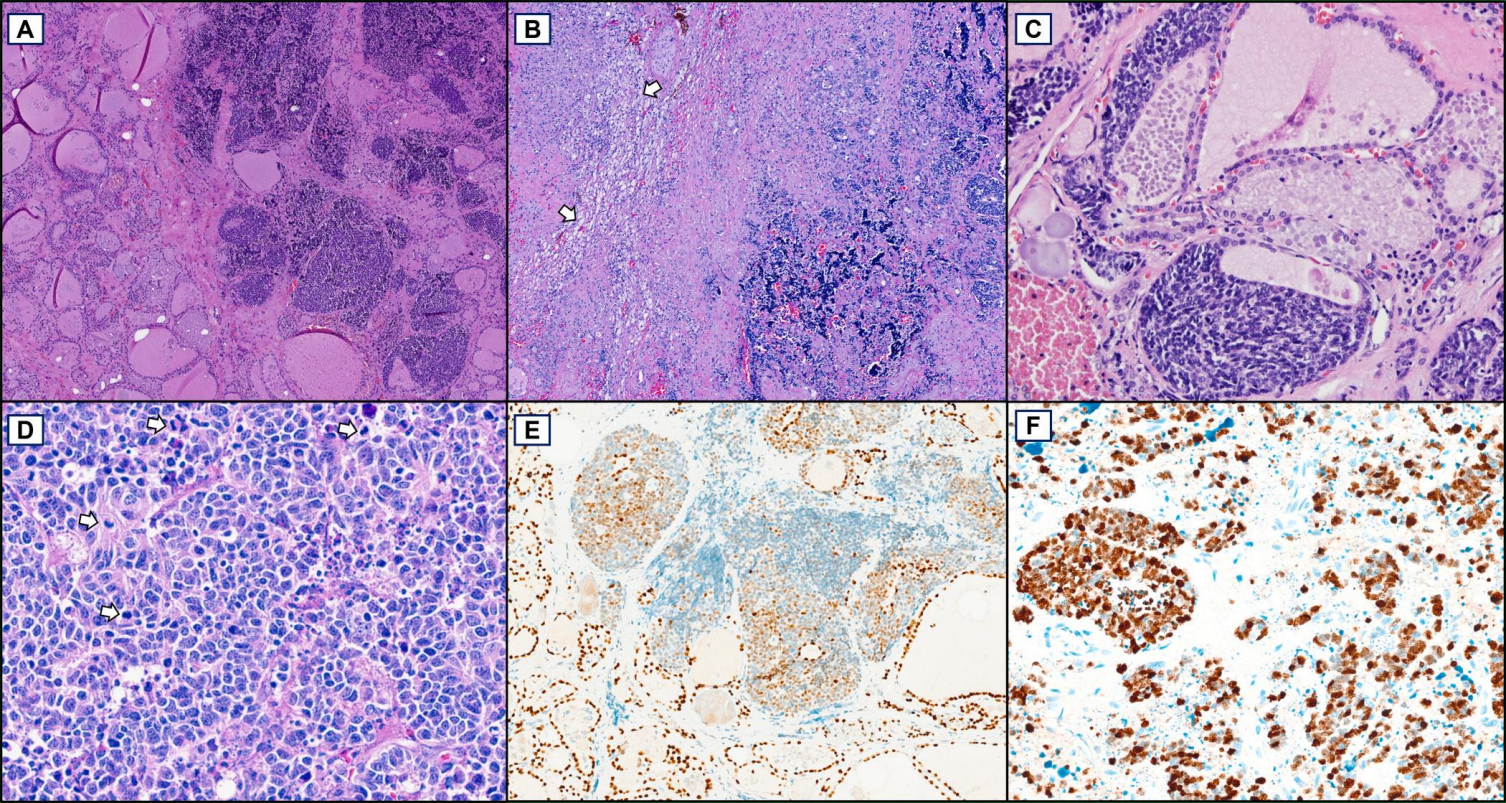
Small cell carcinoma of the thyroid infiltrating the thyroid gland (A, hematoxylin and eosin stain, ×50). Areas of fibrosis and lymphohistiocytic inflammatory infiltrate with frequent foamy macrophages are present (arrows), consistent with treatment effect (B, hematoxylin and eosin stain, ×100). Folliculotropism is also observed (C, hematoxylin and eosin stain, ×200). Tumor cells show a high nuclear‐to‐cytoplasmic ratio and hyperchromatic nuclei with frequent apoptotic bodies and mitotic figures, some of which are indicated by arrows. Focal trabecular arrangements and rosette‐like structures are also observed, supporting neuroendocrine differentiation (D, hematoxylin and eosin stain, ×400). Immunohistochemical study shows focal immunoreactivity for TTF‐1/NKX2‐1 (E, ×200) and a high Ki‐67 proliferation index, estimated at approximately 80%–90% in the most proliferative viable tumor areas after neoadjuvant chemotherapy (F, ×400).

All surgical margins were involved by tumor, and metastatic involvement with extranodal extension was identified in all lymph node compartments examined.

Immunohistochemically, the neoplastic cells showed diffuse positivity for CK8/18, CD56, and synaptophysin, and focal positivity for AE1/AE3, chromogranin A, CEA, and TTF‐1/NKX2‐1 (Figures 3 and 4). A wild‐type p53 staining pattern was observed. The tumor cells were negative for CT, thyroglobulin, PAX8, CK20, p63, NUT, and EBER (in situ hybridization) (Figure 4). Immunohistochemical analysis for CT gene‐related peptide (CGRP) was negative (Figure 4). RNA *in situ* hybridization for CGRP showed no detectable CGRP mRNA expression in tumor cells. Scattered non‐neoplastic C cells in areas of preserved residual thyroid parenchyma showed CGRP mRNA expression, serving as an internal positive control. Ki‐67 immunohistochemistry showed a high proliferation index, estimated at approximately 80%–90% in the most proliferative viable tumor areas after neoadjuvant chemotherapy (Figure 3).

**Figure 4:**
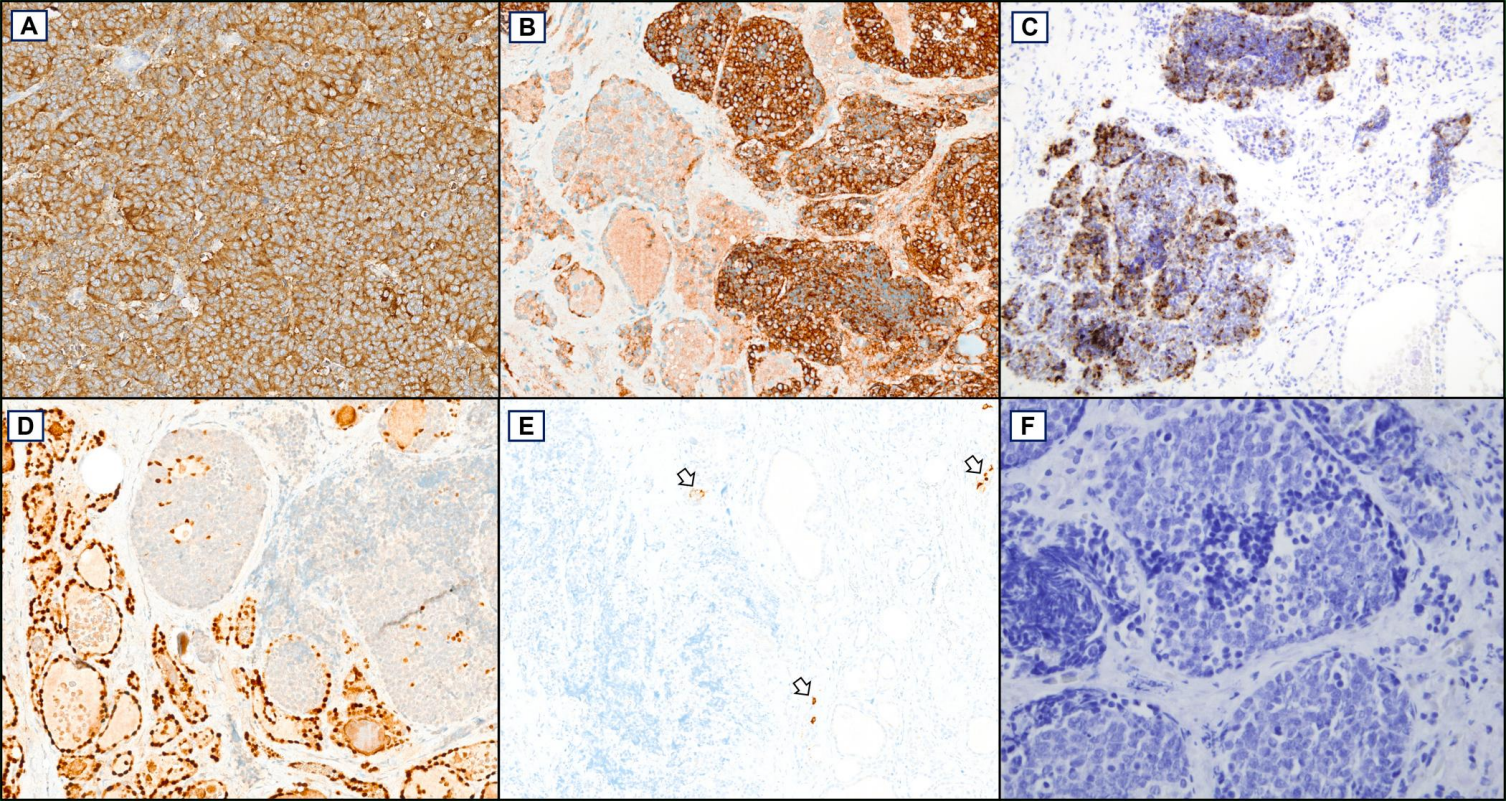
Immunohistochemical study demonstrates immunoreactivity for neuroendocrine markers, namely synaptophysin (A, ×100), CD56 (B, ×200), and chromogranin A; focal immunoreactivity for CEA (C, ×200); and absence of immunoreactivity for PAX8 (D, ×200). Tumor cells are negative for calcitonin (E, ×200), while scattered non‐neoplastic C cells in preserved residual thyroid parenchyma show strong calcitonin immunoreactivity (arrows), serving as an internal positive control. Tumor cells also show no immunoreactivity for CGRP (F, ×400).

Molecular analysis was performed on cytologic material using the Oncomine™ Focus Assay and identified a single *EGFR* exon 3c.345T > A mutation. No mutations were detected in *RET*, *KRAS*, *HRAS*, or *BRAF* genes.

## 4 Discussion

We report a thyroid tumor displaying small cell morphology in the absence of serum CT elevation, amyloid deposition, or immunoreactivity for CT and CGRP, and without molecular alterations typically associated with MTC. The diagnosis was supported by the presence of a highly infiltrative carcinoma composed of small cells with scant cytoplasm, high nuclear‐to‐cytoplasmic ratio, hyperchromatic nuclei, high mitotic activity, apoptotic bodies, tumor necrosis often extensive, and diffuse neuroendocrine marker expression, in keeping with SCC morphology. In contrast, features supporting MTC were lacking, including typical MTC morphology, amyloid deposition, serum CT elevation, CT and CGRP immunoreactivity, and mutations involving genes commonly associated with MTC, such as *RET* and *RAS*. In the absence of histologic, immunophenotypic, and molecular features characteristic of MTC, and with no evidence of metastatic disease at presentation, a diagnosis of primary SCC of the thyroid was favored over a CT‐negative high‐grade MTC. This case highlights both the aggressive clinical behavior and the diagnostic challenges associated with this rare entity.

According to the 5th edition of the WHO Classification of Endocrine and Neuroendocrine Tumours, MTC is classified as a thyroid C‐cell‐derived carcinoma, whereas primary SCC of the thyroid is not recognized as a distinct tumor type [4]. The WHO classification also recognizes high‐grade MTC based on proliferative activity, with high‐grade tumors defined by at least one of the following features: mitotic count ≥ 5 per 2 mm^2^, Ki‐67 proliferation index ≥ 5%, or tumor necrosis [4]. This emphasizes that high‐grade morphology alone does not exclude MTC. Therefore, the main diagnostic challenge in CT‐negative thyroid neuroendocrine tumors with small cell morphology is distinction from CT‐negative high‐grade MTC. In this setting, evidence of C‐cell differentiation, including CT and CGRP expression, amyloid deposition, and molecular alterations involving genes typically associated with MTC, particularly *RET* and *RAS*, is critical.

Because metastatic SCC involving the thyroid is far more likely than a primary thyroid SCC, exclusion of a primary tumor elsewhere, particularly in the lung, is also essential. Other malignant small round cell tumors involving the thyroid should be considered, including NUT carcinoma; thyroblastoma, particularly those cases dominated by primitive small round cell component [5]; Ewing sarcoma and other small round cell sarcomas; and lymphoma. In the present case, extensive clinical and radiologic staging did not identify a primary tumor elsewhere, arguing against metastatic disease, and the remaining diagnostic possibilities were excluded or considered unlikely based on the integrated morphologic, immunohistochemical, and molecular findings.

Only a few cases of CT‐negative thyroid tumors with neuroendocrine differentiation have been reported in the literature, some of which showed small cell morphology. These tumors have been described in the literature, often under different terminologies, such as “calcitonin‐free oat cell carcinoma” [6], “atypical medullary thyroid carcinoma” [7], or “primary high‐grade calcitonin‐negative neuroendocrine carcinoma” [8]. This variability in nomenclature reflects the absence of established diagnostic criteria for primary SCC of the thyroid and the diagnostic overlap with CT‐negative MTC, particularly in tumors showing small cell morphology. To date, only 11 thyroid tumors with small cell morphology in which the available morphologic and immunophenotypic findings did not support a diagnosis of MTC have been reported [6, 8, 9, 10, 11, 12, 13, 14, 15, 16]. Clinicopathological features of the published cases are summarized in Table 1. Similar to our case, most reported tumors exhibited solid, nested, and infiltrative growth patterns without amyloid deposition. Tumor necrosis was present in the majority of cases, whereas vascular invasion was reported in only two cases. Another consistent finding was the absence of CT immunoreactivity, while being consistently positive for neuroendocrine markers. CGRP expression, considered another marker of C‐cell differentiation, has only been evaluated in two cases, both of which were negative, as in our case. Conversely, CGRP positivity in this setting would support C‐cell differentiation and would make a CT‐negative variant of MTC the most likely diagnosis [3].

**Table 1:** Clinicopathological features of reported thyroid small cell carcinomas lacking evidence of medullary thyroid carcinoma and our case.

Ref.	Gender/age (years)	Serum CT	Histological features				Immunohistochemical study							Metastasis	Treatment	Outcome (after diagnosis)
			Growth pattern	Amyloid‐like deposits	Tumour necrosis	Vascular invasion	CT	CGRP	ChA	Syn	CEA	TTF‐1/NKX2‐1	Tg			
[9]	F/82	Normal	Solid Infiltrative	Present	ND	ND	−	−	+	+	+	ND	−	Lymph nodes and distant	Total thyroidectomy	Died (after 23 months)
[6]	F/63	ND	Solid nested/trabeculae Infiltrative	Absent	Present	ND	−	ND	+	+	ND	ND	−	Lymph nodes and distant	Total thyroidectomy	Died (after 1 month)
	M/73	ND	Solid, infiltrative	Absent	Extensive	ND	−	ND	+	+	ND	ND	−	Lymph nodes and distant	None	Died (after 1 month)
[10]	F/59	ND	Solid infiltrative	ND	Extensive	Present	−	ND	+	+	+	+	ND	Lymph nodes	Total thyroidectomy with lymph node dissection and chemotherapy	Died (after 15 months)
[11]^a^	M/64	ND	Nested/nodular	Absent	Present	Present	−	ND	+	+	−	−	+	Lymph nodes	Right hemithyroidectomy with lymph node dissection	No evidence of disease (after 12 months)
[12]	ND/57	Normal	Solid, infiltrative	ND	Present	ND	−	ND	+	+	−	−	−	Distant	Chemotherapy	Alive with disease
[8]	F/42	Normal	Solid	Absent	ND	ND	−	−	−	+	−	+	−	None	Chemotherapy and radiotherapy	No evidence of disease (after 60 months)
[15]	F/56	Normal	Trabecular	ND	Present	ND	−	ND	+	+	−	+	−	Lymph nodes and distant	Total thyroidectomy with lymph node dissection	Alive with disease
[16]	M/33	ND	Solid	ND	ND	ND	−	ND	−	+	−	+	−	Lymph nodes	Total thyroidectomy with lymph node dissection and chemotherapy	No evidence of disease (after 17 months)
[13]	F/40	Normal	Solid	ND	ND	ND	ND	ND	ND	+	ND	+	ND	Lymph nodes and distant	Left hemithyroidectomy with lymph node dissection, chemotherapy and radiotherapy	Alive with disease
[14]^b^	M/34	ND	Solid	Absent	ND	ND	−	ND	−	+	−	−	ND	Distant	Total thyroidectomy with lymph node dissection	Died (after 12 months)
Our case	F/50	Normal	Solid infiltrative	Absent	Present	Present	−	−	+	+	+	+	−	Lymph nodes and distant	Total thyroidectomy with lymph node dissection and chemotherapy	Died (after 10 months)

Abbreviations: CEA, carcinoembryonic antigen; CGRP, calcitonin gene‐related peptide; ChA, chromogranin‐A; CT, calcitonin; F, female; M, male; ND, no data; Syn, synaptophysin; TTF‐1/NKX2‐1, thyroid transcription factor‐1; Tg, thyroglobulin.

^a^ Case reported in the literature as radiation‐associated with a history of previous left thyroid lobe resection.

^b^ Primary small cell thyroid carcinoma combined with poorly differentiated thyroid carcinoma.

Similar to most previously reported cases, our tumor demonstrated focal positivity for TTF‐1/NKX2‐1. Although not specific, CEA expression is considered to favor C‐cell differentiation [2]. Only two previously reported cases demonstrated CEA positivity [9, 10]. However, both TTF‐1/NKX2‐1 and CEA can be expressed in tumors arising from other anatomical sites, particularly pulmonary neuroendocrine carcinomas. Therefore, careful interpretation of TTF‐1/NKX2‐1 and CEA is required, and exclusion of metastatic disease is essential before establishing a diagnosis of primary thyroid SCC [3].

Molecular analysis in our case revealed a single *EGFR* exon 3c.345T > A mutation. No somatic mutations were detected in *RET*, *HRAS*, *KRAS*, or *BRAF* genes. Molecular data on these tumors remain scarce. Among the previously reported similar cases, molecular analysis was performed in only two [11, 14], and neither identified alterations in genes typically associated with MTC. Mussazhanova et al. [11] reported a radiation‐associated SCC of the thyroid with no somatic *RET* mutations, but with microsatellite abnormalities involving the *PTEN* locus, suggesting that PTEN functional loss may have contributed to tumorigenesis. Unlike that case, the present patient had no history of prior therapeutic neck irradiation. In the present case, PTEN expression was retained by immunohistochemistry; however, retained PTEN immunoreactivity does not exclude underlying *PTEN* genetic alterations or functional impairment, particularly in the setting of missense mutations [17]. The most comprehensive molecular analysis reported to date was performed by Kim et al. [14], who described a combined SCC and poorly differentiated thyroid carcinoma. Shared mutations in *ATRX*, *TP53*, and *MYCL* were identified in both components, supporting a common clonal origin, whereas additional alterations, including a *CDK2* mutation and *PDGFRA* and *KIT* amplifications, were restricted to the small cell component. No alterations in genes classically associated with MTC were identified.

Concomitant inactivation of *TP53* and *RB1* is a molecular hallmark of pulmonary SCC, occurring in more than 90% of cases [18]. In contrast, extrapulmonary small cell neuroendocrine carcinomas appear to show a lower frequency of *TP53* molecular alterations and a more heterogeneous molecular landscape; in a recent series, *TP53* molecular alterations were detected in 39.8% of extrapulmonary small cell neuroendocrine carcinomas [19]. Although our molecular panel did not include *TP53* or *RB1*, the tumor showed a wild‐type p53 immunohistochemical pattern, making an underlying *TP53* alteration less likely, but not excluding it [19]. *RB1* status could not be assessed.

The clinical course of the cases in the literature has been variable, with different therapeutic approaches and different outcomes. Some cases demonstrated rapid disease progression and early death, as observed in our patient, whereas others remained alive with or without evidence of disease. Overall, SCC of the thyroid appears to be associated with a poor prognosis [20], similar to that observed in anaplastic thyroid carcinoma [21] and significantly worse than that typically seen in MTC [4].

Primary SCC of the thyroid is an exceptionally rare neoplasm, with only a limited number of cases reported in the literature. Its histogenesis remains controversial, largely due to the morphologic and immunophenotypic overlap with CT‐negative variants of MTC. Given the WHO classification of MTC as a C‐cell‐derived carcinoma, one possibility is that at least some reported CT‐negative thyroid tumors with small cell morphology represent unusual high‐grade variants of MTC. However, the absence of C‐cell lineage markers and MTC‐associated molecular alterations in selected cases raises the possibility of a non‐C‐cell origin. Thyroglobulin and thyrotropin receptor expression was confirmed by quantitative real‐time polymerase chain reaction in a radiation‐associated small cell neuroendocrine carcinoma of the thyroid [11]. In this regard, the molecular findings reported by Kim et al. suggest that, at least in a subset of cases, thyroid SCC may arise through clonal progression from a follicular‐cell‐derived thyroid carcinoma [14]. In their combined SCC and poorly differentiated thyroid carcinoma, shared genetic alterations in both components supported a common clonal origin, whereas additional alterations restricted to the small cell component suggested acquisition of further molecular events during progression. This is in keeping with the concept that poorly differentiated and anaplastic thyroid carcinomas may develop through dedifferentiation of differentiated thyroid carcinoma [21]. This case highlights the diagnostic challenges posed by CT‐negative neuroendocrine tumors of the thyroid and underscores the importance of a multidisciplinary diagnostic approach integrating morphologic, immunohistochemical, molecular, and clinical data. Further studies are necessary to better clarify the histogenetic origin of thyroid SCC, particularly once a relationship with MTC has been excluded.

## Author Contributions

João Carlos Almeida conceived and designed the study. João Carlos Almeida and Patrícia Andrea‐Ferreira were responsible for clinical data acquisition and analysis. João Carlos Almeida, Catarina Costa, Pedro Amoroso Canão, Manuel Sobrinho‐Simões, and José Manuel Cameselle‐Teijeiro contributed to histopathological evaluation and interpretation. João Carlos Almeida drafted the manuscript and prepared the figures. Catarina Costa, Pedro Amoroso Canão, Elisabete Rodrigues, Manuel Sobrinho‐Simões, and José Manuel Cameselle‐Teijeiro critically revised the manuscript. All authors approved the final version of the manuscript.

## Disclosure

The authors have nothing to report.

## Ethics Statement

This study was conducted in accordance with the Declaration of Helsinki of 1975. Consent for research was obtained, and all identifying information was removed to ensure patient anonymity.

## Conflicts of Interest

The authors declare no conflicts of interest.

## Data Availability

The authors have nothing to report.
